# Endomiocardiofibrose como Causa Rara de Transplante Cardíaco e Associação com Trombofilia: Relato de Caso

**DOI:** 10.36660/abc.20210040

**Published:** 2022-01-01

**Authors:** Laura Caroline Tavares Hastenteufel, Nadine Oliveira Clausell, Francine Hehn de Oliveira, Santiago Alonso Tobar Leitão, Lívia Adams Goldraich

**Affiliations:** 1 Hospital de Clínicas de Porto Alegre Serviço de Cardiologia Porto Alegre RS Brasil Hospital de Clínicas de Porto Alegre – Serviço de Cardiologia, Porto Alegre, RS – Brasil; 2 Hospital de Clínicas de Porto Alegre Serviço de Patologia Porto Alegre RS Brasil Hospital de Clínicas de Porto Alegre – Serviço de Patologia, Porto Alegre, RS – Brasil

**Keywords:** Endomiocardiofibrose/transplante, Transplante do Coração, Trombofilia, Protrombina, Insuficiência Cardíaca, Embolia Pulmonar, Trombose Venosa, Mortalidade

## Introdução

A endomiocardiofibrose (EMF) é uma doença rara de etiologia desconhecida caracterizada pela deposição de tecido fibroso no endomiocárdio afetando um ou ambos os ventrículos e o aparelho valvar atrioventricular. O aumento da rigidez e o diâmetro gradualmente reduzido do ventrículo envolvido levam a um padrão restritivo, que se apresenta como insuficiência cardíaca, eventos tromboembólicos, arritmias não fatais ou, menos frequentemente, morte súbita cardíaca.^1^ Embora estudos anteriores relatem baixos índices de sobrevida em pacientes com EMF, em séries contemporâneas, o prognóstico é incerto.^2^ Fatores genéticos, imunológicos, infecciosos e ambientais, entre outros, foram propostos para explicar sua patogênese. Entretanto, não há consenso sobre uma teoria causal unificada.^2,3^ Apesar de seus prognósticos ruins e das opções terapêuticas modificadoras da doença limitadas, a endocardectomia sendo uma delas como medida paliativa,^4^ há uma escassez de dados na literatura que relatem o transplante cardíaco como alternativa terapêutica para pacientes com EMF. Neste estudo, descreve-se o desfecho favorável de uma paciente com EMF que foi submetida a transplante cardíaco. No presente caso, também houve uma associação de EMF com o fator V de Leiden e a mutação do gene da protrombina como parte da apresentação fenotípica, que não havia sido relatada anteriormente.

## Relato de Caso

Uma paciente do sexo feminino de 36 anos da Região Sul do Brasil foi encaminhada ao ambulatório de insuficiência cardíaca devido a um diagnóstico preliminar de EMF, que não é endêmica na região. O histórico médico incluía uma trombofilia hereditária, com testes positivos para fator V de Leiden e mutação do gene da protrombina (20210G>A), associada a eventos tromboembólicos recorrentes, incluindo trombose venosa profunda, tromboembolismo pulmonar e acidente vascular cerebral cardioembólico. O histórico familiar não apresentava indícios significativos de doenças cardíacas. Os exames de laboratório revelaram contagem de eosinófilos levemente elevada, e testes diagnósticos abrangentes não demonstraram má nutrição, doenças autoimunes ou infecções parasíticas. O ecocardiograma transtorácico de linha de base demonstrou fração de ejeção ventricular esquerda preservada e câmaras esquerdas dilatadas com espessura normal de parede. Foram observados padrão de enchimento restritivo e obliteração apical marcada. A paciente relatou limitação funcional leve durante atividades rotineiras.

**Figura 1:**
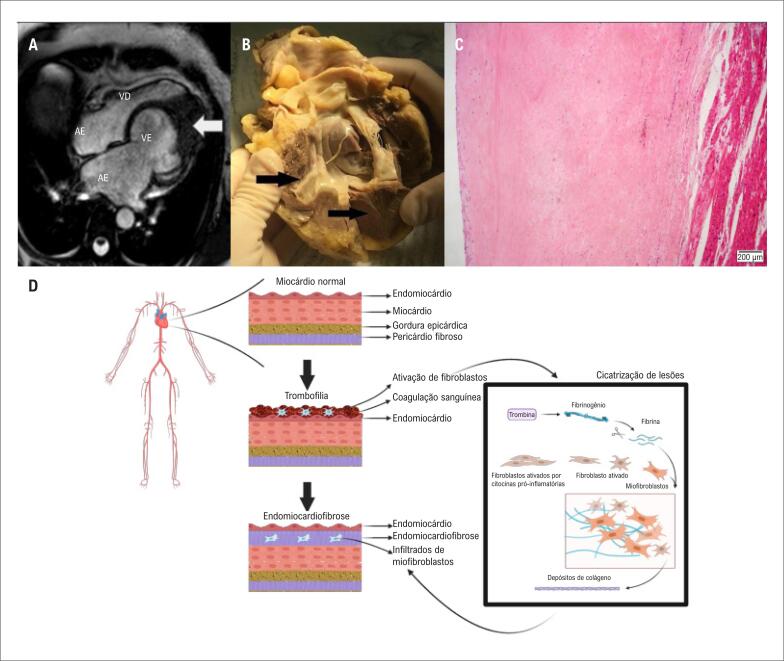
(A) Ressonância magnética cardíaca de quatro câmaras mostrando câmaras esquerdas dilatadas e obliteração cardíaca do ápiice marcada (seta). (B) Vista macroscópica do coração explantado mostrando espessamento endocárdico aumentado mais proeminente nas câmaras esquerdas (setas). (C) Microscopia do coração explantado mostrando uma área de espessamento fibroso grave na camada subendotelial. HE 10X. (D) Mecanismo proposto de trombofilia como contribuidora para a endomiocardiofibrose: o estado pró-trombótico pode promover a ativação de fibroblastos e a deposição de colágeno, que progressivamente se organizam em endomiocardiofibrose (criado com BioRender.com). AE: átrio esquerdo; VE: ventrículo esquerdo; AD: átrio direito; VD: ventrículo direito.

Depois de um período de 16 anos de estabilidade clínica com tratamento médico, que incluía inibidores de enzima conversora da angiotensina, betabloqueadores e anticoagulantes (varfarina), na idade de 52 anos, ficaram evidentes o status funcional de piora progressiva, fibrilação atrial e necessidade crescrente de diuréticos. O ecocardiograma revelou fração de ejeção ventricular esquerda de 45% e acinesia do vértice do ventrículo esquerdo; algumas áreas apicais sugeriam calcificação ou fibrose no endocárdio, compatível com o avanço da EMF; não foi observada valvulopatia significativa, e a função ventricular direita e o diâmetro eram normais. Esses aspectos também foram destacados por imagens por ressonância magnética cardíaca (Figura 1A), mostrando câmaras esquerdas dilatadas e obliteração do ápice ventricular. Um teste de exercício cardiopulmonar demonstrou um volume máximo de oxigênio gravemente prejudicado de 8,4 mL/min/Kg, e a avaliação hemodinâmica invasiva mostrou um índice cardíaco baixo de 1,9 L/min/m^2^. A paciente foi colocada na lista de transplante em seguida. O transplante cardíaco foi realizado sem complicações perioperatórias após um período de sete meses na lista de espera. A análise do coração explantado revelou espessamento endocárdico difuso de até 3 mm com áreas de calcificação distrófica nos dois-terços inferiores do ventrículo esquerdo (Figura 1B). O exame histopatológico mostrou depósito abundante de colágeno extracelular causando grave espessamento fibroso subendocárdico (Figura 1C).

No acompanhamento de 50 meses após o transplante cardíaco, a paciente não apresentava evidência de EMF recorrente em ecocardiogramas de rotina ou biópsias endomiocárdicas de vigilância. Ocorreram três episódios de rejeição celular de moderada a grave no primeiro ano após o transplante, mas todos eles foram tratados com sucesso com corticosteroides. Devido à neurotoxicidade associada ao tacrolimo, a imunossupressão de manutenção consistia em ciclosporina, everolimo e prednisona. Em relação à trombofilia, o regime de anticoagulação foi trocado de varfarina para anticoagulante oral direto. Não se observou recorrência de eventos tromboembólicos desde o transplante.

## Discussão

O presente relato ilustra o curso progressivo da EMF até doença cardíaca de fase terminal em que a indicação do transplante cardíaco no momento certo e o desfecho favorável pós-procedimento sugerem que ele deveria ser considerado uma alternativa terapêutica para pacientes selecionados. Além disso, a associação com a trombofilia hereditária é uma observação interessante, já que os mecanismos subjacentes à evolução da EMF ainda são pouco entendidos.

Como os fatores ambientais e laboratoriais ainda precisam explicar as interações complexas que dão origem à EMF na variedade de cenários em que ela se desenvolve, hipóteses adicionais merecem passar por análises exploratórias posteriores. No presente caso, a associação entre a EMF e a trombofilia levantou a hipótese de que a EMF pudesse ser uma consequência da organização de eventos sucessivos de trombose intracardíaca que levam a fibrose e contração das trabéculas ventriculares. Na verdade, já se propôs que um estado pró-trombótico tenha um papel na patogênese da EMF^5,6^ Shaper e Wright descreveram uma prevalência de 47% de trombos intracardíacos em mais de cem autópsias em pacientes com EMF.^3^ Kartha et al.,^6^ relataram uma possível associação entre deficiência qualitativa e/ou quantitativa de proteína C e a ocorrência de EMF.^6^ Além disso, esse padrão de envolvimento cardíaco foi descrito no contexto de outros possíveis estados pró-trombóticos e/ou doenças autoimunes, tais como a síndrome de Behçet.^7,8^ Como nossa paciente não era de uma região altamente endêmica de EMF nem tinha doenças autoimunes ou infecciosas comórbidas, especula-se que poderia haver uma interação causal entre a trombofilia como um estado pró-trombótico e a EMF (Figura 1D).

Considerando os vários cenários clínicos em que a EMF é identificada, o tratamento precisa abordar, sempre que necessário, a causa primária e o manejo dos sintomas. Alguns pacientes podem se beneficiar de tratamentos imunossupressores quando uma síndrome autoimune estiver presente. Entretanto, a maioria dos pacientes ainda não tem um tratamento específico para sua doença. O tratamento cirúrgico na forma de endocardectomia seguido pelo reparo da válvula mitral e/ou da válvula tricúspide pode melhorar os desfechos, especialmente em centros altamente especializados, embora com limitações. Entretanto, essa abordagem é baseada principalmente em séries de casos e em ensaios não randomizados.^1,2,9^ Moraes et al.,^10^ sugerem a endocardectomia como procedimento paliativo, já que ela não altera o curso progressivo da doença.^10^ No presente caso, como decisão da equipe de cardiologia, levando em consideração a experiência limitada com esse procedimento cirúrgico, que provavelmente traria desfechos diferentes em comparação a centros com altos volumes, decidiu-se realizar o transplante. Na realidade, pacientes selecionados que avançam para doença cardíaca em fase terminal devido à EMF podem se beneficiar do transplante cardíaco com desfecho favorável no curto prazo, especialmente aqueles que não têm doenças sistêmicas subjacentes.^11,12^

## Conclusão

Este relato de caso teve o objetivo de destacar a perspectiva de resultados favoráveis após o transplante cardíaco em pacientes selecionados com EMF. Além disso, os achados podem sugerir uma nova relação mecanística entre EMF e trombofilia hereditária, ainda que sejam necessários estudos para explicar seu papel no escopo amplo de cenários clínicos que podem transcorrer com a EMF.
