# Supplementary material for: Endomiocardiofibrose como Causa Rara de Transplante Cardíaco e Associação com Trombofilia: Relato de Caso
Source: Arq Bras Cardiol. 2022 Jan 1;118(1):103–5. [Article in Portuguese] doi: 10.36660/abc.20210040 (PMC8959054; doi:10.36660/abc.20210040)
Supplement: Translation

# Translation: Endomyocardial Fibrosis as a Rare Cause of Heart Transplantation and its Association with Thrombophilia: A Case Report

**Authors:** Laura Caroline Tavares Hastenteufel, Nadine Oliveira Clausell, Francine Hehn de Oliveira, Santiago Alonso Tobar Leitão, Lívia Adams Goldraich

**Affiliations:** 1 Porto Alegre RS Brazil Hospital de Clínicas de Porto Alegre – Serviço de Cardiologia, Porto Alegre, RS – Brazil; 2 Porto Alegre RS Brazil Hospital de Clínicas de Porto Alegre – Serviço de Patologia, Porto Alegre, RS – Brazil

**Keywords:** Endomyocardiol Fibrosis/transplantation, Heart Transplantation, Thromphilia, Prothrombin, Heart Failure, Pulmonary Embolism, Venous Thrombosis, Mortality

## Introduction

Endomyocardial fibrosis (EMF) is a rare disease of unknown etiology characterized by endomyocardial fibrous tissue deposition affecting either one or both ventricles and the atrioventricular valve apparatus. Increased stiffness and progressively reduced diameter of the involved ventricle lead to a restrictive pattern, which appears as heart failure, thromboembolic events, non-fatal arrhythmias, or less frequently, sudden cardiac death.^1^. Though earlier studies report poor survival rates among EMF patients, in contemporary series prognosis, it is uncertain.^2^ Genetic, immune, infectious, and environmental factors, among others, have been proposed to explain its pathogenesis; however, there is no consensus on a unified causal theory.^2,3^ Despite its poor prognosis and limited disease modifying therapeutic options, endocardectomy among them as a palliative measure,^4^ there is a paucity of data in the literature reporting heart transplantation as a therapeutic alternative for patients with EMF. The present study describes the favorable outcome of a patient with EMF who underwent heart transplantation. In the present case, there was also an association of EMF with both factor V Leiden and prothrombin gene mutation as part of the phenotypic presentation, which has not been previously reported.

## Case Report

A 36-year-old female patient from the South of Brazil was referred to our outpatient heart failure clinic due to a primary diagnosis of EMF, which is not endemic in the region. Past medical history included a hereditary thrombophilia, with positive tests for factor V Leiden and prothrombin gene (20210G>A) mutation, associated with recurrent thromboembolic events, including deep venous thrombosis, pulmonary thromboembolism, and cardioembolic stroke. Family history was unremarkable for cardiac diseases. Laboratory tests revealed normal to mildly elevated eosinophil count and a comprehensive diagnostic workup failed to demonstrate malnutrition, autoimmune diseases, or parasitic infections. Baseline transthoracic echocardiogram showed a preserved left ventricular ejection fraction and dilated left chambers with normal wall thickness; restrictive filling pattern and marked apical obliteration were noted. The patient reported mild functional limitation during ordinary activity.

After a 16-year period of clinical stability on medical therapy, which included angiotensin-converting enzyme inhibitor, beta blocker, and anticoagulant (warfarin), at 52 years of age, progressive worsening functional status, atrial fibrillation, and increasing diuretic requirements were evident. Echocardiography revealed left ventricular ejection fraction of 45% and akinesia of the left ventricular apex; some apical areas were suggestive of calcification or fibrosis in the endocardium, compatible with progression of EMF; no significant valvulopathy was noted, and right ventricular function and diameter were normal. These aspects were also highlighted by cardiac magnetic resonance imaging (Figure 1A), showing dilated left chambers and apex obliteration. Cardiopulmonary exercise testing demonstrated a severely impaired peak oxygen uptake of 8.4 mL/min/Kg, and invasive hemodynamic assessment showed a low cardiac index of 1.9 L/min/m². The patient was subsequently listed for transplant. Heart transplantation was performed without perioperative complications after a seven-month period on the waiting list. Analysis of the explanted heart revealed diffuse endocardial thickening up to 3 mm with dystrophic calcification areas in the inferior two-thirds of the left ventricle (Figure 1B). Histopathologic examination showed an abundant extracellular collagen deposit causing severe subendocardial fibrous thickening (Figure 1C).

**Figure 1:** (A) Four-chamber cardiac magnetic resonance showing dilated cardiac left chambers and marked cardiac apex obliteration (arrow). (B) Macroscopic view of the explanted heart showing increased endocardial thickening more prominent in left chambers (arrows). (C) Microscopy of the explanted heart showing an area of severe fibrous thickening in the subendothelial layer. HE 10X. (D) Proposed mechanism for thrombophilia as a contributor to endomyocardial fibrosis: the prothrombotic state may promote fibroblast activation and collagen deposition, with progressive organization into endomyocardial fibrosis (created with BioRender.com). LA: left atrium; LV: left ventricle; RA: right atrium; RV: right ventricle.

At 50 months of follow-up after heart transplantation, the patient showed no evidence of recurrent EMF on routine echocardiograms or surveillance endomyocardial biopsies. Three episodes of moderate to severe cellular rejection occurred in the first year post-transplantation, but all of them were successfully managed with corticosteroids. Due to neurotoxicity, associated with tacrolimus, maintenance immunosuppression consisted of cyclosporine, everolimus, and prednisone. Regarding the thrombophilia, the anticoagulation regimen has been switched from warfarin to direct oral anticoagulant; no recurrence of thromboembolic events have been noted since transplantation.

## Discussion

The present report illustrates the progressive course of EMF to end-stage heart disease in which the timely indication of heart transplantation and the favorable post procedure outcome suggest it could be considered as a therapeutic alternative for selected patients. Further, the association with hereditary thrombophilia is an interesting remark, as the mechanisms underlying the development of EMF are still poorly understood.

As environmental and laboratorial factors have yet to explain the complex interactions that originate EMF in the variety of scenarios in which it develops, additional hypotheses deserve further exploratory analysis. In the present case, the association between EMF and thrombophilia raised the hypothesis that EMF could be a consequence of the organization of successive intracardiac thrombosis, leading to fibrosis and contraction of the ventricular trabeculae. In fact, a prothrombotic state has already been proposed to play a role in the pathogenesis of EMF.^5,6^ Shaper and Wright described a prevalence of 47% of intracardiac thrombi in more than one hundred autopsies from patients with EMF.^3^ Kartha et al.^6^ reported a possible link between qualitative and/or quantitative protein-C deficiency and EMF occurrence.^6^ Furthermore, this pattern of cardiac involvement has been described in the context of other potential prothrombotic states and/or autoimmune conditions, such as Behcet's syndrome.^7,8^ As our patient was not from a highly endemic region for EMF, nor did she have comorbid infectious or autoimmune conditions, it was speculated that there could be a causal interaction between thrombophilia as a prothrombotic state and EMF (Figure 1D).

Considering the diverse clinical scenarios in which EMF is identified, treatment must address, whenever possible, the primary cause and symptom management. Some patients can benefit from immunosuppressive therapies when an autoimmune syndrome is present; however, most patients lack a specific treatment for their condition. Surgical treatment in the form of endocardectomy, followed by mitral and/or tricuspid repair, may improve outcomes, especially in highly specialized centers, although to a limited extent; however, this approach is mostly based on case series and on non-randomized trials.^1,2,9^ Moraes et al.^10^ suggests endocardectomy as a palliative procedure, as it does not modify the progressive course of the disease.^10^ As a heart team decision, taking into consideration our limited experience with this surgical procedure, which would most likely bring different outcomes when compared to high volume centers, we decided to proceed with transplantation. Indeed, selected patients who progress to end-stage heart disease due to EMF may benefit from heart transplantation with a favorable short-term outcome, particularly those with no underlying systemic diseases.^11,12^

## Conclusion

This case report aimed to highlight the perspective of favorable outcomes after heart transplantation in selected patients with EMF. Furthermore, our findings may suggest a novel mechanistic relationship between EMF and inherited thrombophilia, yet studies are necessary to explain its role in the broad scope of clinical scenarios that may run in line with EMF.
